# The impact of lifestyle modifications, diet, and vitamin supplementation on natural fertility

**DOI:** 10.1186/s40738-015-0003-4

**Published:** 2015-07-25

**Authors:** Gretchen Garbe Collins, Brooke V. Rossi

**Affiliations:** Department of Obstetrics and Gynecology, University Hospitals/ Case Western Reserve School of Medicine, 1000 Auburn Drive, Suite 310, Beachwood, OH 44122 USA

**Keywords:** Infertility, Fertility, Lifestyle, Diet, Stress, Exercise

## Abstract

**Background:**

Infertility is a relatively common condition. When patients are confronted with this diagnosis, there are medical, psychological, and financial sequelae. Patients often wonder if there is anything they can do to optimize their natural fertility or increase the effectiveness of infertility treatments.

**Findings:**

If there is a clear impact on fertility, such as with smoking and alcohol, cessation should be advised. Similarly, weight loss should be recommended if the BMI is in the overweight and obese category, and weight gain should be recommended for an underweight BMI. The evidence surrounding other lifestyle modifications is less clear. There are conflicting data regarding an optimal fertility diet and consumption of vitamins and supplements. Antioxidants seem to improve semen parameters in men, but the effect on female fertility is less clear. If conflicting evidence exists, such as with caffeine consumption or exercise, moderation should be emphasized. Finally, the diagnosis of infertility and subsequent fertility treatments are stressful for both partners. The psychological aspects should not be ignored and methods such as yoga and cognitive behavioral therapy may be beneficial.

**Conclusion:**

Continued research will determine the optimal lifestyle modifications to achieve pregnancy.

## Introduction

Infertility is defined as the inability of a couple to conceive after 1 year of attempting pregnancy. According to the CDC, in the United States, 10.9 % of women aged 15–44 have impaired fertility [1]. In non-mandated states (states in the US that offer minimal or no insurance coverage for fertility treatments), many patients face expensive medical tests and treatments. Patients and physicians are often curious if there are lifestyle modifications to improve their fertility. This article will focus on the evidence surrounding common lifestyle factors such as weight, exercise, substance use, diet, vitamin and antioxidant supplementation, and stress and their effects on fertility. A summary table of each lifestyle and the associated relative risk is provided (Table 1).

**Table 1:** List of lifestyle/consumption and their risk for infertility

Lifestyle	Risk for Infertility
Weight (2)	
BMI 25–39 or <19	RR- 2.2 (95 % CI 1.6-3.2)
Exercise (8)	
<15 min/day	OR- 0.5 (95 % CI 0.3-0.9)
16-30 or 30–60 min/day	OR- 0.3 (95 % CI 0.2-0.5)
Exercise to exhaustion	OR- 2.3 (95 % CI 1.2-4.5)
Tobacco (18)	
Smokers versus Nonsmokers	OR- 1.6 (95 % CI 1.34-1.91)
Alcohol (27)	
High consumers	RR- 1.59 (95 % CI 1.09-2.31)
Low consumers	RR- 0.64 (95 % CI 0.46-0.90)
Diet and Nutrition (38)	
Red Meats	RR- 1.42 (95 % CI 0.98-2.06)
Chicken/Turkey	RR- 1.53 (95 % CI 1.12-2.09)
Processed Meats	RR- 1.04 (95 % CI 0.67-1.62)
Fish	RR- 1.04 (95 % CI 0.64-1.70)
Low Fat Dairy (41)	
2-4 servings/week	RR- 1.32 (95 % CI 0.83-2.11)
5-6 servings/week	RR- 1.77 (95 % CI 1.06-2.94)
High Fat Dairy	
2-4 servings/week	RR- 0.91 (95 % CI 0.67-1.24)
5-6 servings/week	RR- 0.77 (95 % CI 0.52-1.15)
Caffeine (48)	
(>5 cups coffee/day or >500 mg)	OR- 1.45 (95 % CI 1.03-2.04)

## Findings

### Weight

Body mass index (BMI) and weight are closely related to reproductive function. In a study investigating lifestyle factors, time to conception increased in both overweight (BMI >35 kg/m^2^) and underweight (BMI <19 kg/m^2^) individuals. After adjusting for age, menstrual status and other lifestyle variables, compared to women with a normal weight, women with a BMI <19 or ≥ 25–39 kg/m^2^ had a relative risk of time to conception >12 months of 2.2 (95 % CI 1.6-3.2) [2]. Given this, it is recommended that women who are overweight or obese lose weight and women who are underweight should gain weight to improve fertility.

Higher BMI is also associated with negative outcomes for patients undergoing in vitro fertilization (IVF). In a study of 233 IVF cycles, a BMI consistent with being overweight (BMI 25–29.9 kg/m^2^) or obese (BMI ≥ 30 kg/m^2^) was associated with a lower pregnancy rate (23 % and 22 %, respectively) compared with women of a BMI of 20–22.4 kg/m^2^ (pregnancy rate- 42 %) [3]. Similarly, a BMI of ≥ 25 kg/m^2^ has a lower rate of blastocyst formation compared to women with a BMI of <25 kg/m^2^ (54.9 versus 43.9 %, p < 0.007) [4].

Obesity does not seem to have the same impact on male fertility. In a study of 483 men, BMI was unrelated to sperm concentration, motility, or morphology. It was only when the BMI was ≥ 35 kg/m^2^ that there was lower total sperm count than normal weight men (a difference of 86 million sperm) [5]. Similarly, a prospective study of 172 IVF cycles demonstrated no statistically significant associations between men's BMI and pregnancy rate and live birth rate among couples undergoing conventional IVF [6]. However, in a study where couples required IVF with ICSI, male obesity was related to lower odds of having a live birth. Among the couples undergoing ICSI, the odds of a live birth in couples with an obese male partner were 84 % (95 % CI, 10 % to 97 %) lower than the odds in couples with male partners of normal BMI [7].

### Exercise

Exercise performed in different amounts and frequencies has varying effects on male and female fertility. The complex relationship between exercise and reproductive potential is likely due to alterations in the hypothalamic-pituitary axis. Extreme exercise may lead to anovulation and infertility, whereas exercise may result in improved ovulation and fertility in anovulatory obese patients. A large Norwegian study found that women who were active daily were 3.2 times more likely to have fertility problems than inactive women (OR = 3.2, 95 % CI 1.3-7.6). Additionally, in women who exercised a moderate amount (either 16–30 min or 30–60 min), the risk of infertility was decreased compared to women who exercised < 15 min a day (OR = 0.3, 95 % CI 0.2-0.5; OR = 0.5, 95 % CI 0.3-0.9 respectively). Exercising to exhaustion was associated with an increased risk of infertility (OR = 2.3, 95 % CI 1.2-4.5) [8]. The Nurses’ Health Study found that vigorous exercise for a minimum of 30 minutes a day was also associated with a decrease in ovulatory disorder infertility [9].

The frequency and amount of exercise also seems to impact outcomes in women pursuing IVF. A prospective study examined the exercise regimens of 2,232 women undergoing IVF. Women who reported regular exercise had a similar live birth rate compared to women who did not exercise (OR = 0.8, 95 % CI 0.7-1.0). Women who exercised 4 or more hours per week for 1–9 years were 40 % less likely to have a live birth (OR = 0.6, 95 % CI: 0.4-0.8), three times more likely to experience cycle cancellation (OR = 2.8, 95 % CI 1.5-5.3), twice as likely to have an implantation failure (OR = 2.0, 95 % CI 1.4-3.1) or pregnancy loss (OR = 2.0, 95 % CI 1.2-3.4) compared to women who did not report exercise. In another study of 2,572 women, there were no differences in IVF outcomes between women who regularly exercised and those who did not. However, compared to women who did not exercise, women who exercised regularly for 1–5 years were at greater risk for failure of cycle stimulation (OR = 1.9, 95 % CI 1.2-3.2), implantation failure (OR = 1.5, 95 % CI 1.1-2.0), and failure to develop a live birth after a chemical pregnancy (OR = 1.9, 95 % CI 1.3-2.8) [10].

In contrast, regular exercise does not appear to affect semen parameters. A prospective study of 2,261 men found that none of the semen parameters were altered with regular exercise, except bicycling >/= 5 hours a week was associated with low sperm concentration (OR = 1.92, 95 % CI 1.03-3.56) and low total motile sperm (OR = 2.05, 95 % CI 1.19-3.56) [11].

### Substance Use

When reviewing substance use studies it is important to remember that substances may be used in congruence with one another (ex: smoking and alcohol), and it may be difficult to tease out the effect of each. Additionally, lifestyle factors and consumption may fluctuate, and many studies only capture a snapshot of a current lifestyle.

#### Tobacco

Approximately 30 % of reproductive age women and 35 % of reproductive age men in the United States smoke cigarettes [12]. The toxins in cigarette smoking have been correlated with subfertility, endocrine disorders, earlier onset of menopause, premature ovarian failure, and decreased implantation rate [13–17]. The early onset of menopause associated with smoking suggests an association with early follicular depletion. According to a meta-analysis, the odds ratio for the risk of infertility in women smokers versus non-smokers was 1.60 (95 % CI 1.34-1.91) [18]. It also showed that smoking results in a delay of conception by more than a year. Similarly, compared with non-smokers, women smokers have decreased ovarian response to hyperstimulation (12.12 +/−5 versus 8.62+/− 4 mature oocytes retrieved), lower clinical pregnancy rate (29.6 vs. 10.0 %), and lower AMH levels (3.86+/−1.92 versus 3.06 +/−1.68 μg/l) [19]. Smoking is also associated with many earlier pregnancy complications including spontaneous miscarriages, bacterial vaginosis, preterm labor, and ectopic pregnancy [18, 20]. However, with smoking cessation, fecundity returns to a similar rate as to non-smokers, even if cessation occurs within one year of attempting conception [21]. For infertile women, simply discussing and providing basic information on the effects of smoking on infertility is highly effective in achieving smoking cessation [22].

There is less consistency in the literature regarding the effects of smoking on male infertility. One of the largest studies evaluated 655 male smokers and 1,131 nonsmokers and found that cigarette smoking was associated with a significant decrease in sperm concentration (millions of sperm/mL of semen) (67.7 ± 65.9 vs. 79.9 ± 75.0, p < 0.01) total sperm count (million) (229.4 ± 251.5 vs. 278.1 ± 264.2, p < 0.01), and total number of motile sperm (million) (105.6 ± 132.7 vs. 126.6 ± 136.8, p < 0.01) [23]. Similar to women, smoking cessation improves fecundity and sperm parameters [24].

#### Alcohol

There are conflicting data regarding the effects of alcohol consumption on female infertility. Whereas some studies quote an improvement in fecundity, others show a detriment or no change. There are many studies that suggest a trend toward increased alcohol consumption and decreased conception [25, 26, 2]. In a large Swedish study of 7,393 women, the risk of infertility was significantly increased in women who were high consumers of alcohol (>140 g alcohol, or 10 drinks, per week) (RR = 1.59, 95 % CI 1.09-2.31) and decreased in women who were low consumers (less than 50 g, or 4 drinks, per week) (RR = 0.64, 95 % CI 0.46–0.90) when compared with women who were moderate consumers (between 50–140 g alcohol per week) [27]. Similarly, in a prospective study of 430 Danish couples seeking first time pregnancy, the odds of conception decreased with increasing alcohol consumption in a dose–related fashion. Compared with non-drinkers, women who consumed 1–5 drinks per week had a fecundity OR of 0.61 (95 % CI 0.04-0.93) and women consuming >10 drinks per week an OR of 0.34 (95 % CI 0.22-0.52) [25]. In contrast, a population-based cohort study of 29,844 Danish women suggested that time to conception was shorter in women who drank wine compared to women that drank no alcohol [28]. An Italian study of 1,769 women found no connection between alcohol consumption and difficulty conceiving [29]. Certainly alcohol consumption should be avoided when pregnant, as no safe level of alcohol consumption has been determined during pregnancy [30].

There does not appear to be an association between alcohol consumption and abnormal semen analysis parameters. Some studies demonstrated a detrimental effect of alcohol on sperm parameters, while others showed no or even a beneficial effect [31–33].

For women desiring IVF, two prospective cohort studies evaluated alcohol consumption prior to the IVF cycle and subsequent cycle outcome. In the first study, an increase in one drink per day among women was associated with 13 % fewer oocytes retrieved [34]. In the women with any documented alcohol consumption, there was a non-significant decreased odds of pregnancy. In men, alcohol use was not associated with a change in semen parameters. The strongest association between men’s alcohol intake, pregnancy, and spontaneous abortion was seen when the consumption occurred closest to the time of semen sample collection. The second study had differing findings. This study found that in women who consumed at least four drinks/week there was a decreased live birth rate as compared to those who drank less than four drinks a week (OR = 0.84, 95 % CI 0.71-0.99) [35]. Couples in which both partners drank at least 4 drinks per week had a decreased likelihood of live birth compared to couples in which both drank less than 4 drinks per week (OR = 0.79, 95 % CI 0.66-0.96).

### Diet & Nutrition

The optimal fertility diet has yet to be established, and the effects of nutritional factors on fertility are largely unknown. However, dietary modifications have been shown to improve ovulatory disorder infertility. Three studies specifically evaluated the relationship between protein intake and ovulatory disorder infertility. Two of the studies were randomized trials that compared low (15 % of energy) versus high protein (30 % of energy) diets. The studies did not note any difference in ovulation or circulating hormone levels of androgens between the two groups [36, 37]. The third study was a large, prospective study of 18,555 women. The consumption of red meats and chicken/turkey was associated with a greater rate of ovulatory disorder infertility (RR = 1.42, 95%CI 0.98-2.06; RR = 1.53, 95%CI 1.12-2.09), whereas the consumption of processed meats and fish was not associated with a greater rate of ovulatory disorder infertility (RR = 1.04, 95%CI 0.67-1.62; RR = 1.04, 95%CI 0.64-1.70, respectively). Consumption of foods rich in vegetable protein showed a decreased risk of ovulatory disorder infertility, but it did not reach statistical significance [38].

In an effort to establish a fertility diet, the diets of a cohort of 17,544 women were obtained and the reproductive outcomes were followed. These data were obtained from the Nurses’ Health Study II, a prospective cohort study which started in 1989 and obtained information through a questionnaire given to registered nurses aged 25–42. Based on the dietary intake and reproductive outcomes of these women, a high fertility diet consists of:

- Low intake of trans-fat, with a simultaneous greater intake of monounsaturated fat
- Low intake of animal protein with greater vegetable protein intake
- High intake of high-fiber, low glycemic carbohydrates
- Greater preference for high-fat dairy products
- High non-heme iron intake (mostly found in plant foods)

The women who adhered to this type of diet had a 66 % (RR = 0.34, 95 % CI 0.23–0.48) lower risk of ovulatory disorder infertility and a 27 % (RR = 0.73, 95 % CI 0.57-0.95) lower risk of infertility due to other causes [9]. In men, semen parameters may be improved by diets consisting of limited processed meats and cheese, and by increased low-fat dairy and fish [39].

#### Dairy products

The relationship between dairy consumption and ovulatory disorder infertility has also been studied. Dairy food intake has been associated with a lower risk of insulin resistance and ovulatory dysfunction [40]. However, data from the Nurses’ Health Study II found contradictory results. An increase in low-fat dairy foods by 1 serving per day (calorie count was held constant) was associated with an 11 % greater risk of ovulatory disorder infertility (95 % CI 2-21 %). For low-fat dairy foods, the relative risk for ovulatory disorder infertility was 1.32 (95 % CI 0.83-2.11) for women consuming 2–4 servings per week and 1.77 (95 % CI 1.06-2.94) for women consuming 5–6 servings per week. For high-fat dairy foods, the relative risk was 0.91 (95 % CI 0.67-1.24) for women consuming 2–4 servings per week and 0.77 (95 % CI 0.52-1.15) for women consuming 5–6 servings per week [41].

#### Soy and isoflavonoids

Soy may help female fertility, yet hinder male fertility. There is a clear relationship between the consumption of soy with low sperm concentration. Soy contains isoflavones that bind to the estrogen receptor and have mild estrogenic activity [42, 43]. It is theorized that this mild estrogenic activity may affect spermatogenesis, induce capacitation and a premature acrosome reaction, or lower testosterone levels; however, these findings are from animal studies and the data are inconsistent [44]. Human data from a cross-sectional study of 99 men demonstrated dietary intake of soy and isoflavones were inversely related to sperm concentration. Consumption of soy and isoflavones had more of an impact on semen parameters of men with normal or above normal sperm concentrations [45]. Other foods that contain phytoestrogens include walnuts, soybeans, cereals (oats, barley, wheat, and rice), legumes, berries, apples, carrots, ginseng, fennel, and anise used to make bourbon and beer [46].

In contrast to men, women may actually benefit from consumption of phytoestrogens during infertility treatments. A prospective cohort study evaluated live birth rates at different amounts of soy isoflavone consumption [47]. Compared to those with no soy isoflavone consumption, the odds ratios of live births for women consuming 0.54-2.63 BMI/d was 1.32 (OR = 0.76-2.27, 95 % CI 0.76-2.27), 1.87 (OR = 1.12-3.14, 95 % CI 1.12-3.14) for women consuming 2.64-7.55 mg/d, and 1.77 (OR = 1.03-3.03, 95 % CI 1.03-3.03) for women consuming 7.56-27.89 mg/day. For reference, a 3 oz serving of tofu has 20 mg isoflavones.

#### Caffeine

Daily caffeine consumption is very common among reproductive-aged couples. The role that caffeine plays in fertility is not well-defined. In a large multi-centered European retrospective study, consumption of high levels of caffeine (>5 cups of coffee/day or >500 mg) was associated with an increased odds of subfertility (defined as time to pregnancy 9.5 months or more) (OR = 1.45, 95 % CI 1.03-2.04) [48]. In contrast, a prospective Danish study of 3,628 women evaluated the relationship between time to pregnancy and caffeinated beverages, and found no significant association between caffeine consumption (coffee, tea, soda) and fecundity [49]. Similarly, a cohort study of women without a history of infertility followed them for 8 years to evaluate the effect of caffeine on ovulatory disorder infertility [50]. Caffeine intake did not increase the risk of ovulatory disorder infertility, and there was no association between caffeinated coffee, decaffeinated coffee or tea and infertility.

There are also varying results among women undergoing assisted reproductive technology (IVF). In a cohort study of 619 women undergoing in-vitro fertilization (IVF), caffeine intake did not reduce overall pregnancy rates, however a reduction in the number of retrieved eggs was observed [51]. These findings are consistent with a larger, prospective study that demonstrated that compared with women who do not drink caffeine, the likelihood of live birth was not significantly different for women who drank low (0–800 mg/week; OR = 1.00, 95 % CI = 0.83–1.21), moderate (>800–1400 mg/week; OR = 0.89, 95 % CI = 0.71–1.12), or high levels of caffeine (>1400 mg/week; OR = 1.07, 95%CI = 0.85–1.34). Typically there are roughly 95 mg of caffeine in an 8-ounce cup of coffee. Neither the type of caffeinated drink (tea, soda, coffee) nor men’s caffeine intake had a significant effect on live birth rate. However, increasing caffeine use had a significantly lower peak estradiol level, but no difference in number of oocytes retrieved, fertilization or implantation rate [52].

The American Society for Reproductive Medicine states that moderate caffeine consumption (one to two cups of coffee per day or its equivalent) before or during pregnancy has no apparent adverse effects on fertility or pregnancy outcomes [30].

### Vitamins & Supplements

#### Antioxidants

Antioxidants act to reduce the amount of reactive oxygen species, including hydroxyl radicals, superoxide anions, and hydrogen peroxide [53]. When there is an imbalance of reactive oxygen species there may be an increase in sperm DNA structural damage, as well as an unclear link to female infertility [54]. Commonly studied supplemental and dietary antioxidants include vitamin D, vitamin E, vitamin C, β –carotene, and coenzyme Q10.

A study from of 1,192 infertile women found that 68.6 % of them were vitamin D insufficient (<32 ng/mL), which is higher than the average population [55]. Ozkan et al. performed a small prospective study that demonstrated women with higher vitamin D levels (34.4 vs. 25.6 ng/mL) in their serum and follicular fluid had a higher clinical pregnancy rate following IVF [56]. However, other studies did not confirm this result [57, 58]. Franasiak et al. performed a retrospective cohort study of 517 women undergoing IVF, and found that pregnancy rates did not differ when comparing different serum vitamin D levels (<20, 20–29.9, and ≥30 ng/mL) [59]. Similarly, in a prospective pilot study of infertile men, the incidence of low vitamin D was 76.9 % (mean serum vitamin D level 23.6 ng/mL). After treatment with vitamin D supplements, the rate of low sperm motility (<40 %) improved [60]. Food sources high in vitamin D levels include eggs, fatty fish, dairy, and cod liver oil.

Vitamin E also appears to be associated with positive reproductive outcomes. Vitamin E supplementation (20 mg) in women ≥35yo has been associated with a shorter time to pregnancy (HR = 1.07, 95%CI 1.01-1.13) [61]. Vitamin E supplementation in men may also help to decrease oxidative damage to sperm and improve motility. A study of 20 men randomized to vitamin E and selenium or vitamin B-complex for 3 months resulted in increased motility among subjects taking vitamin E and selenium, but not with the vitamin B [62]. However, recommendations for vitamin E and selenium supplementation should be done cautiously as high-doses of these supplements have been associated with increased incidence of prostate cancer, cardiac complications, and breast cancer in women. There is also an increase in mortality at doses greater than 400 IU/day [63]. Food sources high in vitamin E include sunflower seeds, almonds, spinach, papaya, and dark leafy greens.

The role of vitamin C and β -carotene supplementation on infertility is less clear and depends on the BMI. Ruder et al. performed a secondary analysis of a randomized controlled trial to determine whether antioxidant intake decreased the time to pregnancy in women with unexplained infertility [64]. While consumption of antioxidants decreased time to pregnancy, it was dependent on BMI and the antioxidant. A significantly shorter time to pregnancy was seen in women with BMI < 25 kg/m^2^ with increasing vitamin C (HR = 1.09, 95 % CI 1.03–1.15) and in women with BMI ≥ 25 kg/m^2^ with increasing β -carotene (HR = 1.29, 95 % CI 1.09–1.53). The optimal daily amount of each antioxidant has not yet been suggested.

In men, varying amounts of vitamin C and β –carotene supplementation may be associated with slight improvements in semen parameters. The Rochester Young Men’s study was a cross-sectional study of male reproductive health that assessed dietary intake and semen quality in healthy men. Increased β-carotene (total daily (supplement and diet): >6,059 μg/d vs. <2,520 μg) and lutein (total daily: > 4,480 μg vs. <1,734) intake were associated with a 6.5 % higher progressively total motile sperm count. Lycopene consumption (total daily: >13,508 vs. <4,808 μg) was positively associated with a 1.7 % higher rate of morphologically normal sperm [54]. Vitamin C intake has varying results depending on the dose and the study performed. Daily doses of 200 mg and 1000 mg have been shown to lower sperm agglutination [64, 65], but in the Rochester Young Men’s study there was a dose dependent improvement in semen quality that corresponded to a total intake of about 148 mg/day [54]. More prospective studies clearly need to be completed to determine the exact required doses of the antioxidants and the effect on male semen analysis parameters.

Coenzyme Q10 is a component of the mitochondrial respiratory chain that is required for energy metabolism. CoQ10 is an antioxidant, an energy promoting agent, a membrane stabilizer, and a regulator of mitochondrial permeability transition pores. Given these actions, it is theorized that this added cellular energy may improve sperm count and motility in men and egg quality in women. In men, the amount of CoQ10 in the seminal fluid shows a direct correlation with semen parameters. A strong correlation between sperm count, motility and ubiquinol (reduced from of CoQ10) has been reported [66, 67]. Reduced levels of CoQ10 were noted in infertile men with idiopathic and varicocele-associated asthenospermia [68]. Supplementation with CoQ10 (200 mg/day twice daily) was studied in this population, and after 6 months of treatment there was an increase in seminal plasma, sperm cells, and sperm cell motility [69]. In a randomized trial between placebo and 300 mg coenzyme Q10 supplementation, the men who were supplemented with the CoQ10 had improved sperm count (57.8 vs. 47.8 million, p < 0.01), motility (27.6 vs. 23.1 %, p < 0.01), and morphology (9.6 vs. 7.8 %, p < 0.07) [70]. The benefits for CoQ10 supplementation in women are less clear. Mouse models have shown improvement in fertility and oocyte quality in old mice when treated with CoQ10 [71]; however, this model has yet to be repeated and no human studies have been performed to support this data. A study of women with diminished ovarian reserve treated with dehydroepiandrosterone (DHEA) with or without the addition of CoQ10 found no benefit in the addition of CoQ10 [72]. There is likely minimal harm in taking CoQ10 [73], but there is not strong evidence to recommend taking it at this point to improve fertility. Food sources with CoQ10 include seafood and meats, but is difficult to obtain through the diet as there are not many foods that contain CoQ10.

#### Folate

Folate is required for the synthesis of DNA, transfer RNA, cysteine, and methionine, which are required during periods of rapid cell growth. Given the peri-conceptional period is a time of cellular growth, it was postulated that folate supplementation may improve reproductive outcomes. In a prospective cohort study of 232 women, live birth rates in women undergoing IVF were 20 % higher (95 % CI 8-31 %) among women with the highest amount of supplemental folate intake (>800 mcg/day) compared to women taking the lowest amount (<400 mcg/day). This study also suggested that folate supplementation was superior to dietary folate [74]. Similarly, a prospective cohort study of women undergoing IVF in the Netherlands found that a doubling in the follicular folate level was associated with three-fold increase in pregnancy [75]. From this data, women should be advised to take 800 mcg/day of folate during fertility treatments and throughout pregnancy.

#### Dehydroepiandrosterone (DHEA)

Testosterone stimulates follicular development in the presence of adequate gonadotropin concentrations. DHEA may act as a pre-hormone of ovarian testosterone and therefore may provide another treatment option. The role of DHEA in fertility was also proposed when Gleicher et al. studied women with diminished functional ovarian reserve (defined as abnormally high Follicle Stimulating Hormone levels and/or abnormally low anti-mullerian hormone) [76]. They found that total testosterone levels were lower in these women compared to similar age-matched infertile women without diminished ovarian reserve. In one study of 38 women over the age of 40 planning to start IVF treatment, 25 mg DHEA was administered for 12 weeks prior to starting IVF medications. In this group there were 10 spontaneous and 9 ongoing pregnancies [77]. Unfortunately the same encouraging results were not seen when women undergoing IVF were treated with DHEA. A study of 31 infertile women who were treated with DHEA supplementation (25 mg three times a day) starting 2 months prior to an IVF cycle found no improvement in total number of metaphase II oocytes (4.45 ± 0.47 vs. 2.09 ± 0.26), fertilization (3.65 ± 0.49 vs. 2.0 ± 0.27), Grade I embryos (1.52 ± 0.25 vs. 0.55 ± 0.18), pregnancy rate (30 % vs. 9.1 %) and live birth rate (25 % vs. 0 %) compared to the 2 prior cycles without DHEA supplementation [78]. There is currently an ongoing randomized controlled trial in England to evaluate the ovarian response in women with poor ovarian reserve undergoing IVF treated DHEA versus placebo [79]. There are minimal data about women with infertility taking DHEA who are not undergoing IVF. Furthermore, there are no proposed male benefits to DHEA supplementation.

### Psychological Stress

Stress can have physical, mental, and emotional manifestations. Significant physical or psychological stress, such as anorexia nervosa, alters reproductive function, but the evidence regarding the effects of moderate stress is mixed [80]. Louis et al. studied the effects of stress on infertile couples by measuring salivary α- amylase, which has been found to increase during stressful stimuli. Alpha- amylase elevation corresponded with a poorer probability of conception [81]. However, a study of 2,695 women with infertility found no difference in pregnancy outcomes depending on the self-proclaimed stress level prior to treatments [82]. A meta-analysis including 14 studies on stress, did not find a worse outcome in women with higher levels of distress [83].

Physical, in-person, emotional support still seems to be superior to support through the internet and chat rooms, but the use of the internet for support can still help people facing infertility by educating, empowering, and diminishing feelings of depression [84]. Anxiety and stress respond well to treatments such as cognitive behavioral therapy as well as mind-body interventions. Yoga is one method of stress management and relaxation that may be beneficial for women undergoing fertility treatments. Yoga helps with relaxation through meditation, breathing, and achieving a deep sense of calmness. This may help the woman communicate more clearly with physicians, maintain a healthier lifestyle, and be patient through the sometimes long and cumbersome fertility treatments [85]. Mind-body therapy that focuses on cognitive behavior therapy, relaxation training, negative health behavior modification, and social support components was found to improve pregnancy rates for those undergoing their second IVF treatment cycle (52 % vs. 20 % pregnancy rate, p < .05) [86]. The stresses of infertility affect male partners as well, and this stress may manifest as erectile dysfunction [87]. Offering men similar stress reducing treatment options may also be effective in treating their stress.

## Conclusions

It is the role of the physician to help the patient identify modifiable risk factors, especially given the cost and time commitment associated with infertility treatments. If patients are made aware of simple lifestyle changes that could impact their fertility, they are generally receptive to making those adjustments. All patients should be encouraged to strive for a healthy, sustainable lifestyle. In areas where there is clear evidence that a lifestyle behavior may impact fertility, such as smoking, the patient should be encouraged to modify behavior. However, many lifestyle changes are difficult to study, and when the recommendations are less clear, the limitations of evidence should be discussed. Exercising, as well as alcohol and caffeine consumption, should be performed in moderation. More studies on the effects of stress on infertility are also providing information about treatment options such as cognitive behavioral therapy and yoga. Making these lifestyle modifications may aid in achieving the ultimate goal of a healthy pregnancy.

## Abbreviations

BMI: Body Mass Index
DHEA: Dehydroepiandrosterone
IVF: In vitro Fertilization
